# Randomized Single-Case Experimental Designs in Healthcare Research: What, Why, and How?

**DOI:** 10.3390/healthcare7040143

**Published:** 2019-11-13

**Authors:** René Tanious, Patrick Onghena

**Affiliations:** Faculty of Psychology and Educational Sciences, Methodology of Educational Sciences Research Group, KU Leuven—University of Leuven, 3000 Leuven, Belgium; patrick.onghena@kuleuven.be

**Keywords:** single-case experimental designs, visual analysis, effect sizes, randomization tests

## Abstract

Health problems are often idiosyncratic in nature and therefore require individualized diagnosis and treatment. In this paper, we show how single-case experimental designs (SCEDs) can meet the requirement to find and evaluate individually tailored treatments. We give a basic introduction to the methodology of SCEDs and provide an overview of the available design options. For each design, we show how an element of randomization can be incorporated to increase the internal and statistical conclusion validity and how the obtained data can be analyzed using visual tools, effect size measures, and randomization inference. We illustrate each design and data analysis technique using applied data sets from the healthcare literature.

## 1. Introduction

“Averaging data across many subjects can hide a multitude of sins: The experimental treatment may fail to affect the behavior of some subjects, and may even lead to contrary effects in others. As a consequence, statistically significant results based on large sample sizes are not persuasive.” [1]. Most people trust their healthcare providers with adequate care of a large variety of symptoms, ranging from a simple cold to more complex health problems, such as chronic pain, chronic diseases, or allergies. The opening quote by Perone highlights that rigorous experimental testing and persuasive results are needed to justify the patients’ trust in their healthcare providers. Perone also highlights the individuality of each person when it comes to finding an effective treatment, which by definition limits the applicability of large-scale group studies for situations in which symptoms are highly idiosyncratic in nature.

Following this logic, a shift towards individualized testing and experimental results in healthcare followed the accumulation of evidence that general healthcare diagnoses and interventions often fail to accurately describe and relieve patient symptoms (e.g., [2,3,4]). Already 25 years ago, McHorney and Tarlov recognized that the available health status surveys at the time were not adequate for individual patient monitoring in clinical practice [5]. McHorney and Tarlov reviewed five available health status measures and concluded that “the most problematic feature of the five surveys was their lack of precision for individual-patient applications. There was little evidence of the validity of the five surveys for screening, diagnosing, or monitoring individual patients. At this time, however, it seems that new instruments, or adaptation of existing measures and scaling methods, are needed for individual-patient assessment and monitoring”. Similarly, finding effective treatments for individual patients is difficult to achieve without individually tailored interventions. Turk argued in his influential paper on customizing pain treatments that a substantial proportion of patients does not benefit from generic treatments in spite of an increasingly better understanding of the mechanisms of pain [4]. According to Turk, limited success of chronic pain treatments at the time was caused by assuming homogeneity between patients, which Turk labeled as the “patient and treatment uniformity myths”. In Turk’s view, the remedy to this myth is a better matching of empirical data to patient characteristics in order to design individual treatment plans.

These limits of group studies, specifically in healthcare, were recognized in clinical practice after decades, in which they were thought to be the gold standard and the “*N*-of-1 randomized controlled trial” was included among the highest levels of evidence in the Oxford Centre for Evidence-Based Medicine and in the Evidence-Based Medicine Guidelines of the American Medical Association [6,7,8]. Outside the medical field, *N*-of-1 randomized controlled trials have already long been in use under the name single-case experimental designs (SCEDs). In this paper, we review and empirically demonstrate the use of a specific form of individualized experimentation: randomized SCEDs. Vohra [9] accurately summarized a shift towards SCEDs in healthcare and evidence-based medicine, when she said that “although evidence-based medicine has embraced large parallel group trials as the gold standard for health research, there are limitations in the ability to apply data from these trials into routine clinical practice. Rigorous research methods that yield high-quality data from individual patients have the opportunity to not only inform the care of that individual, but also the group of individuals who suffer from the same condition(s). Rather than starting with a group, and extrapolating inferences at the level of the individual, single-case experimental designs (*sic*) evaluate treatment response at the level of the individual, and when combined, may inform how we should treat groups of patients with similar conditions”.

As Vohra explained, SCEDs turn the logic of group studies upside down to find effective treatments in healthcare applications, in which the individual is the unit of analysis and intervention. SCEDs come with the additional advantage that they require fewer resources and are often practically more feasible, for example when many variants of a therapy exist and they cannot all be tested in large-group studies [10].

SCEDs are thus viable and powerful alternatives to group studies in healthcare research. To draw valid conclusions from SCEDs about novel or existing treatments, it is pivotal to choose a strong design and adequate data analysis tools. In this paper, we showcase how an element of randomization can be incorporated into the design of an SCED to strengthen the internal validity of the experiment. We first define SCEDs, distinguish SCEDs from other non-experimental forms of case research, and present a typology of different types of SCEDs. We then move on to define and discuss each type accompanied by an applied publication from the healthcare literature. For each applied data set, we explain stepwise how an element of randomization can be implemented and how the obtained data can be analyzed using visual analysis, effect size calculation, and randomization tests.

## 2. Single-Case Experimental Designs: Definition and Overview of Design Options

Contemporary textbooks on SCEDs follow a long tradition. An early and fierce proponent of SCEDs was B.F. Skinner [11], proclaiming in his 1956 seminal paper on the scientific method that “we are within reach of a science of the individual. This will be achieved, not by resorting to some special theory of knowledge in which intuition or understanding takes the place of observation and analysis, but through an increasing grasp of relevant conditions to produce order in the individual case”.

Excellent introductory texts on SCEDs for healthcare professionals are available in Morgan and Morgan [12,13], who credited Skinner as an important figure in the advancement of SCEDs for behavioral and healthcare sciences. Other recommended textbooks on the methodology of SCEDs include Barlow et al. [14], Kazdin [10], and Ledford and Gast [15]. In spite of the fact that many different (sub-)forms of SCEDs exist, they all have some common underlying features. All the forms of SCEDs comprise of repeated measurements (e.g., daily disability ratings) taken from a single entity (e.g., a pain patient) under different levels of at least one independent variable (e.g., treatment for chronic pain) [16,17]. Table 1 provides a typology of SCEDs with four overarching categories: phase designs, alternation designs, multiple baseline designs, and changing criterion designs (cf. [18,19]). Within each of these categories, different design options exist. For further information about each design example, interested readers are referred to the key references in Table 1.

A systematic review of published SCEDs with health behavior outcomes is available in a study by McDonald et al. [35]. As the authors pointed out, it is important to clearly distinguish these types of single-case experimental research from other types of non-experimental case research. In non-experimental case studies, no intervention takes place. Instead, the behavior of interest is observed and measured over time as it occurs naturally. As such, observational case studies can give valuable insights about how behaviors evolve naturally over time. Contrary to that, the ultimate goal of SCEDs as a form of individualized experimentation is to assess whether a causal relationship exists between the independent and dependent variables. This can give information about which treatment works best for a patient by observing changes in the health outcome behavior under different manipulations of the independent variable (e.g., different therapies for decreasing self-harming behavior in a patient with depressive symptoms). McDonald et al. pointed out that, in experimental forms of single-case research with health behavior outcomes, it is advised to incorporate randomization into the design if possible. In the following paragraphs, we define the concept of randomization, define each type of SCED, present published data sets from the healthcare literature for each type, and show how an element of randomization can be included in each category to strengthen the internal validity and analyze the data using the randomization tests.

## 3. Randomization in Single-Case Experimental Designs

The incorporation of an element of randomization in the design of experiments has a long-standing history and interested readers are referred to Finch [36], Kempthorne [37], and Onghena [38] for an extensive historical discussion of the concept. Randomization is not a prerequisite for conducting an SCED. However, the added value of incorporating an element of randomization in the design of an SCED has been extensively discussed in the literature. It is recommended that an element of randomization should be incorporated whenever the research conditions under consideration allow it to enhance the scientific credibility of SCEDs [39,40,41,42]. Onghena and Edgington further contended that randomized experiments are in many aspects superior to non-randomized experiments due to stronger control over confounding factors, such as time, the participants, or the setting [18]. Furthermore, randomization facilitates some difficult decisions in the planning phase of an SCED. For example, the randomization procedure helps make decisions about the order, in which treatments are presented to a patient [43].

In addition to the advantages that randomization offers in terms of enhanced scientific credibility, control over internal validity threats in the planning of an SCED, randomization has great added value to the statistical conclusion validity. Kempthorne pointed out that randomization in the design of experiments frees the researcher from having to use statistical models, of which assumptions might not actually have been met by the experimental procedure and resulting data [44]. Similarly, Todman and Dugard asserted that the incorporation of randomization in the design of experiments makes such experiments eligible for statistical tests based on the random assignment procedure actually utilized in the experiment [45].

These statistical tests are called randomization tests. Randomization tests are valid and powerful significance tests under the assumption that “in experiments in which randomization is performed, the actual arrangement of treatments … is one chosen at random from a predetermined set of possible arrangements” [24]. Comprehensive textbooks on randomization tests in general and in particular for SCEDs are available in Edgington and Onghena [46] and Todman et al. [21], respectively. The steps involved in conducting a randomized SCED and analyzing the obtained data with a randomization test are explained in Heyvaert and Onghena [47] and Tanious et al. [48]. Briefly, these steps are as follows: hypothesis formulation and determination of the significance level and the number of measurements; determination of the randomization scheme; conduct of the experiment and calculation of the observed test statistic; and obtaining of the reference distribution and *p*-value.

To better understand the concept of randomization in SCEDs, it might be helpful to reconsider how randomization is used in group studies. In group studies, participants are assigned randomly to the different experimental conditions. In SCEDs, where one entity is exposed to all the levels of the independent variable(s), this is per definition not possible. Instead, measurement occasions are randomly assigned to the different levels of the independent variable(s) [18,49]. In research practice, however, oftentimes there are restrictions for this random assignment procedure of measurement occasions to treatments in SCEDs due to ethical or financial reasons. It might, for example, be unethical to withhold treatment from a patient with chronic pain just for the sake of adhering to the randomization scheme. Similarly, the duration of a study and the length of an intervention phase depend, in part, on the financial resources available. If financial resources are limited and the study duration is accordingly short, a fast introduction of the intervention is preferred, which places restrictions on the random assignment procedure. Additionally, the chosen SCED might place restrictions on the random assignment procedure. These restrictions will be discussed per type of SCED in subsequent sections.

## 4. Phase Designs

Phase designs consist of measurements taken in consecutive phases that implement different levels of the independent variable(s). In the terminology of phase designs, “A” stands for baseline measures, i.e., measurements of the dependent variable(s) without any manipulation of the independent variable(s), and “B” stands for experimental measures, i.e., measurements of the dependent variable with the manipulation(s) of the independent variable(s) in place [14,15]. According to the What Works Clearinghouse guidelines [50], three measurements per phase are required to meet the minimum evidence standards and five measurements per phase are recommended to meet evidence standards without reservation for all the designs under the phase category (see also [51,52,53]).

The most basic forms of phase designs are AB and ABA designs. In the former, initial baseline measurements are followed by measurements taken under the manipulation of the independent variable(s). In the latter, the intervention is withdrawn and the B-phase measurements are followed by a second A-phase. The ABA design is often also referred to as the withdrawal design [14]. While both of these designs are initially appealing due to their simplicity, they come with significant drawbacks. Guidelines on the conduct of SCEDs require at least three potential demonstrations of an effect to demonstrate experimental control over a dependent variable (e.g., [50,54]). An effect can be demonstrated with each phase change, and thus the AB design offers one potential demonstration, while the ABA design offers two potential demonstrations. A related concern is that, with few phase changes, any observed effect might coincide with external circumstances [50]. Ledford and Gast summarized the problems associated with the AB and ABA designs as follows:

(a) “The ABA design is more useful than the basic AB design from an experimental perspective. However, you would not select this design at the outset to evaluate intervention effectiveness due to the practical and ethical considerations of terminating a study with a participant in baseline conditions. From a research perspective, if ethically defensible and practical, it would be more appropriate to expand to an ABAB design, thereby replicating the effect of the independent variable on the target behavior.” [15].
(b) As Ledford and Gast pointed out, sometimes practical and ethical reasons render it impossible to implement more intricate designs [15]. Therefore, not to dismiss the AB design altogether, Michiels and Onghena [55] and Onghena et al. [56] discussed techniques for increasing the experimental validity of this design. These techniques include incorporating randomization into the design, colleting a sufficiently large number of data points, and replicating across participants.
(c) As Ledford and Gast further explained, an ABAB design offers one more possibility of demonstrating the effect of an independent variable than an ABA design. With three potential demonstrations of an effect, the ABAB design is therefore the minimum phase design to meet the quality standards. If phase designs implement more than one distinct manipulations of the independent variable, each level is labeled with a distinct letter in alphabetical order. For example, in the ABACA design, two additional levels of the independent variable are present (B and C). It is also possible for two treatments to be administered within the same phase. The use of hyphens between each distinctive phase is then recommended to delineate the phases from one another. For example, in an A-B-A-BC design, intervention B is first administered separately and in the second experimental phase together with intervention C.

**Figure 1:**
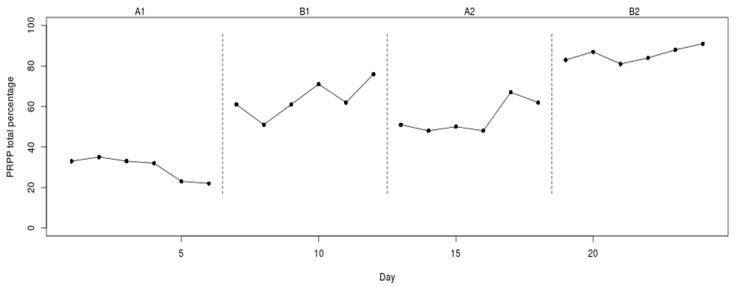
Example of an ABAB design. Data from Nott et al. (2008) [57].

Figure 1 presents the results of an ABAB design used to investigate the effectiveness of occupational therapy with adults demonstrating agitation and post-traumatic amnesia following brain injury [57]. During the A-phases, subjects received daily standard occupational therapy, including systematic instruction, task adaptation, environmental modification, physical guidance, and facilitation. During the B-phases, daily occupational therapy was provided using the Perceive, Recall, Plan and Perform (PRPP) System approach. As Nott et al. explained, the PRPP System is a dynamic intervention process based upon all the stages of information processing [57]. During both phases, the subject’s information processing capacity was measured daily as a percentage score of task performance. Thus, a higher score indicates better information processing by the subject. Figure 1 shows the results of a 35-year-old female with diffuse axonal injury resulting in restlessness, excessive response to external stimuli, poor attention, and memory impairment.

In phase designs, all the data are graphed as one continuous time-series, facilitating the observation of changes over time. Vertical dashed lines indicate phase changes. For all analyses in this paper, we look at the data aspect level, which may be operationalized as the mean score in a given phase [54]. A visual inspection of the means in each phase reveals that there is a noticeable difference in the level of the scores between A1 and B1. This change in level is not reverted when the intervention is withdrawn, even though the level of the scores in A2 is lower than in B1. When the intervention is introduced again (B2), there is a noticeable level change in score when compared to that in the preceding baseline phase (A2).

A randomization test can supplement this visual assessment with a quantification (test statistic) and information about the statistical significance of the effect (*p*-value). The general null hypothesis is that the treatment is ineffective. The researchers expected that the intervention would lead to an increase in information processing. Therefore, we chose this as our one-sided alternative hypothesis. We chose the conventional significance level α of 0.05. To quantify the differences in level, we used the sum of B-phase means minus the sum of A-phase means as our test statistic [58], which can be written as: $\left({\bar{B}}_{1}+\;{\bar{B}}_{2}\right)$ − $\left({\bar{A}}_{1}+{\bar{A}}_{2}\right)$. If a researcher expects the scores to be lower in the B-phases, $\left({\bar{A}}_{1}+{\bar{A}}_{2}\right)-\left({\bar{B}}_{1}+{\bar{B}}_{2}\right)$ might be a more suitable test statistic. Two-sided tests can be performed by using the absolute difference between the sum of the B-phase means and the sum of the A-phase means written as: $\left|\left({\bar{A}}_{1}+{\bar{A}}_{2}\right)-\left({\bar{B}}_{1}+{\bar{B}}_{2}\right)\right|$.

In total, 24 measurements were taken. For the validity of the randomization test, it is important to assume that this had been determined a priori. In a phase design, an unrestricted randomization scheme, in which each measurement can either be A or B, is, per definition, not possible. Therefore, a restricted randomization scheme that takes the phase nature of the design into account should be chosen. Following guidelines on the conduct and analysis of SCEDs, we chose a restricted randomization scheme that allows for at least three measurements per phase. The number of possible ways of randomizing 24 measurements was calculated as: $\left(\begin{array}{c} 24-3\left(3+1\right)+3\\ 3 \end{array}\right)=$ 455, so that each of the four phases contains at least three measurements (cf. 58). Below is a non-exhaustive list of randomizations for illustrative purposes (the experiment, as it was carried out, is marked in bold):

AAABBBAAABBBBBBBBBBBBBBB
AAAABBBAAABBBBBBBBBBBBBB
AAAAABBBAAABBBBBBBBBBBBB
AAAAAABBBAAABBBBBBBBBBBB
        …
**AAAAAABBBBBBAAAAAABBBBBB**
AAAAAAAAAAABBBBBAAAABBBB
AAAAAAAAAAAABBBBAAABBBBB.

**Figure 2:**
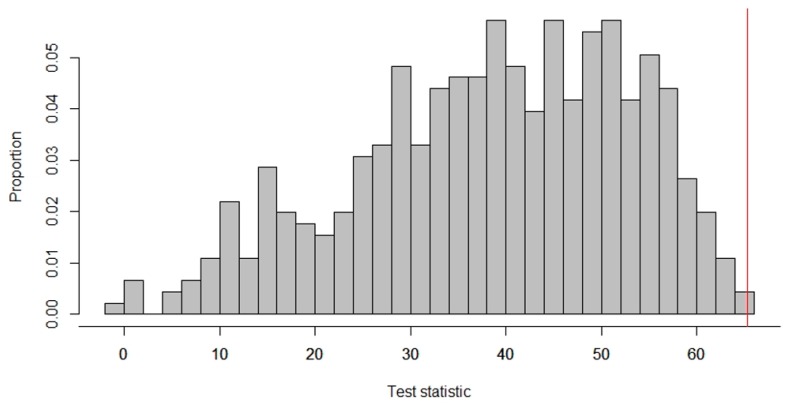
Reference distribution of test statistics for an ABAB design by Nott et al. [57].

The observed test statistic was calculated as: (63.67+85.67)−(29.67+54.33)=65.33, meaning that, on average, task performance by the subject increased by 65.33% during the intervention phases. How does this observed test statistic compare to the test statistics that would have been obtained with the other randomizations? To answer this question, we needed to locate the observed test statistic in the reference distribution of all the test statistics possible, given the randomization scheme (see Figure 2).

The vertical red line indicates the observed test statistic. All the test statistics on this line or further to the right indicate test statistics at least as large as the observed one. In this case, none of the other randomizations would have led to a test statistic as high as or higher than the observed one. The *p*-value of a randomization test equals the number of test statistics as large as or larger than the observed one (when the expected effect is an increase in the dependent variable). Thus, for the data in Figure 2, the *p*-value was calculated as: $\frac{1}{455}=0.002$, and we rejected the null hypothesis that the treatment is ineffective.

## 5. Alternation Designs

Contrary to phases designs, alternation designs do not consist of distinctive phases. Rather, as the name suggests, alternation designs rely on the fast alternation of treatments that are each associated with a distinct stimulus [25]. Another contrast to phase designs is that, in alternation designs, there is no requirement for a minimum number of measurements under the same condition to establish a steady pattern of responding before alternating the treatments [59]. In alternation designs, “A” refers to measurements taken under the first treatment or baseline and “B” refers to measurements taken under the second treatment. If more treatments are tested, each subsequent treatment is labeled with a distinct letter in alphabetical order. The completely randomized design is the simplest design in the alternation category [16,60]. In a completely randomized design, each measurement has an equal chance of being either A or B. As Edgington pointed out, this random assignment procedure is analogous to the random assignment of subjects to treatments in between-group studies [22]. While this design has a strong internal validity, a researcher might end up with an undesirable order of treatments [61]. For example, if a researcher conducts an experiment with 10 measurement occasions to test the effect of a novel exercise therapy for patients with arthritis, one of the possible randomizations would be AAAAABBBBB. Such a design has weak control over internal validity threats such as history and maturation as discussed in the section over AB designs. Alternating treatments designs and randomized block designs rule out such undesirable sequences. In an alternating treatments design, a limit is placed on the maximum number of measurements taken consecutively under the same condition. Recommended limits are two consecutive measures [27,28] or three consecutive measures [14]. In some research situations, however, it is not feasible or undesirable to administer the same treatment twice or three times in a row within a given timeframe. A randomized SCED that takes this constraint into account is the randomized block design. Edgington [22] gave an example of a randomized block SCED by referring to a study of Smith [62]. Smith tested the effectiveness of three different drugs to relieve narcoleptic symptoms over a period of 15 days. Smith divided the 15 days into five segments of three days. During each segment of three days, each drug was administered once and the order of administration was determined randomly with one drug per day. The logic of a randomized block SCED becomes clearer in comparison to the block logic in group studies. In group studies using a randomized block design, participants are allocated randomly to treatment blocks consisting of different conditions, whereas in a randomized block SCED treatments closely together in time are grouped into blocks and the order of treatment administration within each block is randomized [61].

**Figure 3:**
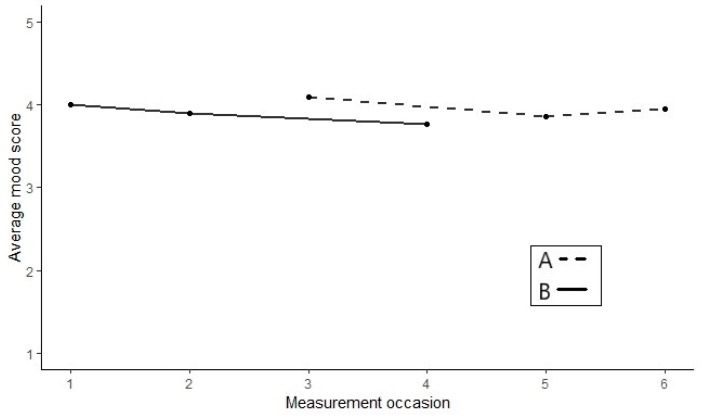
Example of an alternating treatment design. Data from Roumen et al. [63].

Figure 3 presents the results of an alternating treatments design used to examine the effect of androgen supplementation in six healthy oral contraceptive users, who experience mood disturbances during regular oral contraceptive use [63]. Each study phase consisted of one menstrual cycle, during which the subjects continued their regular contraceptive use, supplemented either with a placebo (A-measurements) or with supplemental androgen (B-measurements). For both treatments, daily mood measures were taken with a single item question to be answered on a 5-point Likert scale, where 5 indicated a very positive mood. Figure 3 shows the results of a 23-year-old female who had been an oral contraceptive user for nine years prior to the study.

Whereas in phase designs the data for all treatments are plotted as one continuous time-series, in alternation designs each treatment is plotted as its own time-series. Each data point represents the mean mood score for that menstrual cycle. A visual inspection of Figure 3 reveals that two of the A-measurements are higher—indicating a better mood—than all B-measurements. This is contrary to the expected treatment effect.

For the randomization test, the null hypothesis and significance level remain the same as for the phase design. The alternative hypothesis is that the treatment leads to an increase in mood score. The researchers determined to take six measurements in total for each participant with each treatment for three menstrual cycles. The researchers further determined that the same treatment should not be administered for more than two consecutive menstrual cycles. With these constraints, there are 14 possible randomizations. Below is an exhaustive list of all possible randomizations for illustrative purposes (the experiment, as it was carried out, is marked in bold):

AABABB
AABBAB
ABAABB
ABABAB
ABABBA
ABBAAB
ABBABA
BAABAB
BAABBA
BABAAB
BABABA
BABBAA
BBAABA
**BBABAA**.

**Figure 4:**
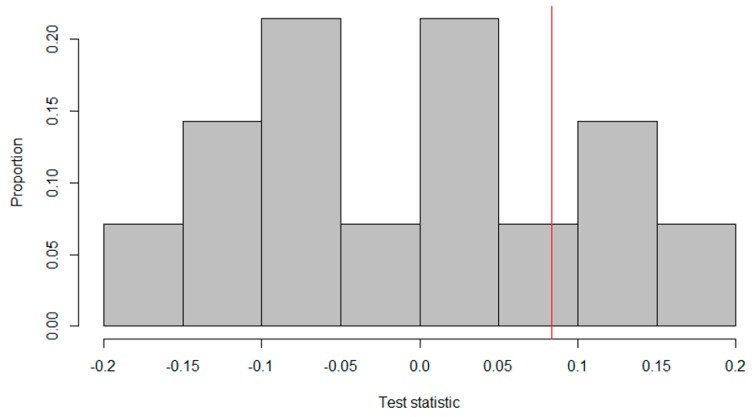
Reference distribution of test statistics for an alternating treatment design by Roumen, et al. [63].

To quantify the difference between the A- and B-measurements, Roumen et al. subtracted the mean of the intervention measurements from the mean of the baseline measurements by using ($\bar{A}-\;\bar{B}$). The observed test statistic for the data in Figure 3 was calculated as: 3.97 − 3.89 = 0.08, meaning that, on average, the subject’s mood score was 0.08 higher on the 5-point Likert scale during the baseline measures. Figure 4 shows the reference distribution for the alternating treatments design.

As the researchers expected the treatment to lead to an increase in mood scores, all the test statistics on the red line or to its left indicate randomizations that would have led to a higher treatment effect. There are 11 randomizations, for which the treatment effect is higher than the observed one. Thus, the *p*-value was calucated as: $\frac{11}{14}=0.79$. Accordingly, we did not reject the null hypothesis that the treatment is ineffective for this subject.

## 6. Multiple Baseline Design

Conceptually, multiple baseline designs are closely related to phase designs. The multiple baseline designs consist of a series of replicated AB designs. The term was coined by Baer et al. [29], “An alternative to the reversal technique may be called the ‘multiple baseline’ technique. This alternative may be of particular value when a behavior appears to be irreversible or when reversing the behavior is undesirable. In the multiple-baseline technique, a number of responses are identified and measured over time to provide baselines against which changes can be evaluated. With these baselines established, the experimenter then applies an experimental variable to one of the behaviors, produces a change in it, and perhaps notes little or no change in the other baselines.”

As Baer et al. [29] pointed out, there are situations, in which a change in a dependent variable is irreversible. For example, if a patient suffering from cardiac arrhythmia receives a pacemaker to reduce his/her feelings of dizziness, the pacemaker cannot simply be removed again to assess whether the feelings of dizziness increase again as a result (as is for example done in an ABAB design). In situations like that, the multiple baseline design provides a valid alternative. Baer et al. first defined the multiple baseline across outcomes design. However, replications in the multiple baseline design can also be established across participants or settings, and the intervention is introduced in a staggered way to the different units [64].

The staggered introduction of the intervention is an important feature for the validity of all variants of the multiple baseline design. Consider a novel therapy designed to reduce feelings of claustrophobia in a patient, who avoids crowds, narrow spaces, and windowless rooms. The staggered introduction of the intervention implies that, while the therapy is applied to the first setting (crowds), the other settings (narrow spaces and windowless rooms) remain in the baseline measures. When the therapy is applied to the second setting (narrow spaces), the third setting (windowless rooms) remains in the baseline measures. Finally, the intervention is introduced to the third setting (windowless rooms). If the feelings of claustrophobia decrease only in the setting to which the therapy is applied, and in the other settings that are in the baseline measures, no changes in feelings of claustrophobia are observed, then the multiple baseline design gives powerful evidence for the effectiveness of the therapy. In general, “the power of such designs comes from demonstrating that change occurs when, and only when, the intervention is directed at the behavior, setting, or subject in question” [14] (p. 202, emphasis in original).

**Figure 5:**
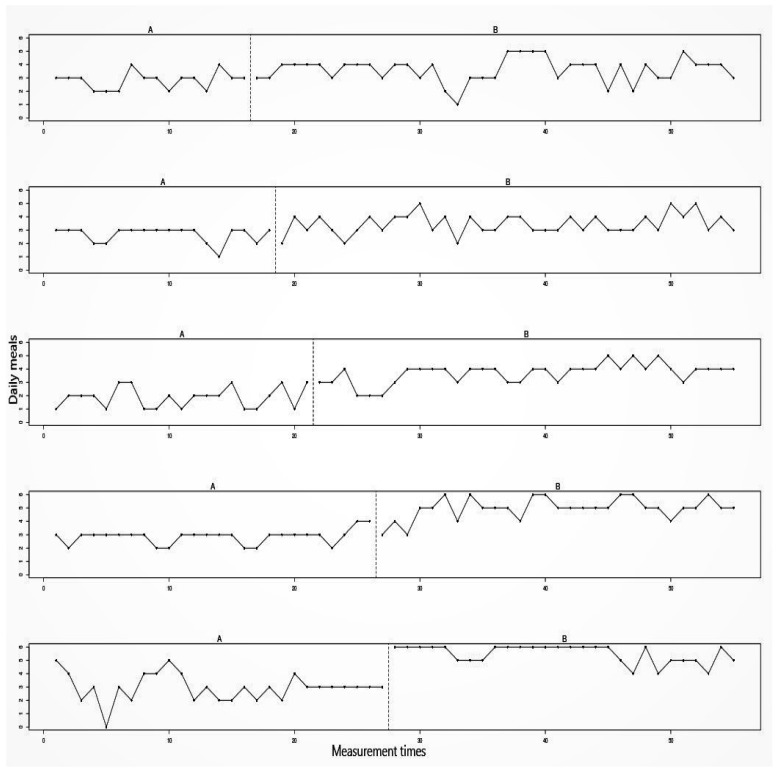
Example of a multiple baseline design across cases. Data from Abrahamsson et al. [65].

Figure 5 shows the results of a multiple baseline design across participants used to investigate the effectiveness of video-based cognitive behavior therapy for treating eating disorders in five patients living far from urban centers [65]. During the baseline phases, subjects registered their daily eating patterns and symptoms of disordered eating. During the intervention phases, subjects received cognitive behavioral therapy sessions via a mobile video application. These sessions focused on establishing a regular meal schedule. Furthermore, subjects were encouraged to regularly record their weight, but these data were not recorded by the researchers. Figure 5 displays the number of daily meals consumed by the subjects, which Abrahamsson et al. [65] chose as the main outcome variable. Subjects self-recorded their daily eating frequency by means of a treatment-specific food diary. The researchers hypothesized that the treatment would lead to a higher frequency of daily meals, indicative of less binge eating.

In a multiple baseline design, each participant, outcome, or setting is plotted as its own time-series. For the first participant, the intervention was introduced on the 17th day; for the second participant, the intervention was introduced on the 19th day; for the third participant, the intervention was introduced on the 22nd day; for the fourth participant, the intervention was introduced on the 27th day; and for the fifth participant, the intervention was introduced on the 28th day. A visual inspection of the graphed data reveals that the magnitude of change after the introduction of the intervention differs between participants. For participants three, four, and five, the increases in level seem higher than for participants one and two.

The null hypothesis and the significance level remain the same as in the previous examples. The alternative hypothesis is that the video-based cognitive behavior therapy leads to a higher frequency of daily meals. A restricted randomization scheme for multiple baseline designs has to take into account the staggered introduction of the intervention across participants, meaning that the intervention cannot start on the same day for more than one participant. Abrahamsson et al. determined a priori that the total duration of the study is 55 days [65]. Furthermore, the researchers determined a priori that the moment of phase change from the baseline to the intervention would occur randomly for each participant between the 15th and 36th day. This randomization scheme was chosen so that each participant has a baseline length of at least two weeks and an intervention phase length of at least 20 days. For the chosen randomization scheme, there are 3,160,080 (calculated by $\frac{22!}{\left(22-5\right)!}$) randomizations that allow for a staggered introduction of the intervention. Below is a non-exhaustive list of baseline phase lengths per participant (the ones actually used in the experiment is marked in bold):

14,15,16,18,30
**16,18,21,26,27**
18,19,21,22,28
22,24,28,30,34
   …
26,29,30,32,35
28,29,30,31,32
29,30,31,32,33
31,32,33,34,35.

**Figure 6:**
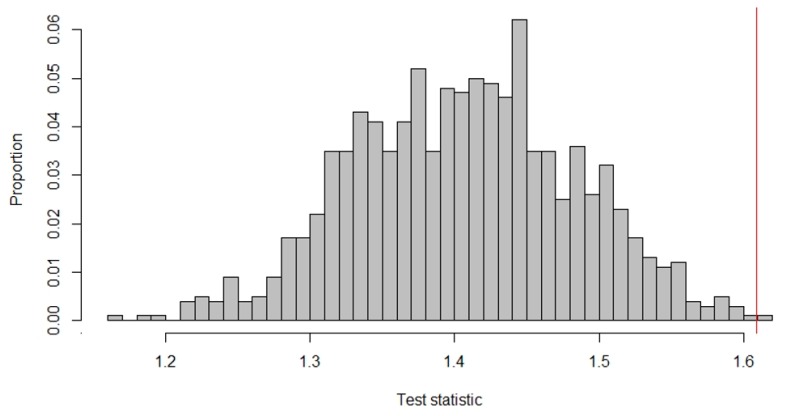
Reference distribution of test statistics for a multiple baseline design i [65].

To quantify the intervention effectiveness, we choose the mean difference between the intervention and the baseline measurements as our test statistic, which can be calculated by $\frac{\left({\bar{B}}_{1}+\;{\bar{B}}_{2}+{\bar{B}}_{3}+{\bar{B}}_{4}+{\bar{B}}_{5}\right)-({\bar{A}}_{1}+{\bar{A}}_{2}+\;{\bar{A}}_{3}+{\bar{A}}_{4}+{\bar{A}}_{5})\;}{5}$. The observed test statistic for the data displayed in Figure 5 was calculated as: $\frac{\left(3.59+3.46+3.68+4.97+5.5\right)-\left(2.81+2.67+1.86+2.85+2.96\right)}{5}=1.61$, meaning that the intervention leads, on average, to an increase of 1.61 in the frequency of daily meals. Given the large number of possible randomizations, it is computationally not feasible to locate the observed test statistic in the reference distribution of all possible randomizations. Therefore, Abrahamsson et al. used a Monte Carlo random sampling procedure [65]. This procedure takes a random sample of 1000 randomizations based on all permissible randomizations. Figure 6 shows how the observed test statistic compared to the test statistics that would have been obtained by the other 999 randomizations.

Two randomizations would have led to a test statistic as large as the observed one or even larger. The *p*-value thus equals  0.002, calculate by $\frac{2}{1000}$, and we can reject the null hypothesis that the treatment is ineffective.

## 7. Changing Criterion Design

The last category in the typology of SCEDs is the changing criterion design. This design was first introduced and demonstrated by Hartmann and Hall [31]. In the changing criterion design, a criterion that the subject has to meet is set. This criterion changes constantly between adjacent phases to systematically decrease or increase the frequency of the dependent variable. After the initial baseline measures, treatment is never withdrawn [43]. Barlow et al. argued that the lack of treatment withdrawal throughout the course of the study is a major strength of the changing criterion design and that this feature makes the design especially attractive for clinical studies, for example in the treatment of dangerous behaviors such as self-harming [14]. Klein et al. elaborated that the changing criterion design is especially valuable in situations, in which an immediate, abrupt increase or decrease in a dependent variable may be difficult to achieve or undesirable [32]. Furthermore, the stepwise changes in the frequency of the dependent variable may facilitate habitual changes in a subject’s behavior [66]. In their seminal paper, Hartmann and Hall conducted two experiments using the changing criterion design. In the first study, Hartmann and Hall used a reward strategy to increase the number of math problems correctly solved by a behaviorally disordered boy. In the second study, a financial incentive strategy was used to stepwise reduce the number of cigarettes smoked by a heavy smoker. Hartmann and Hall [31] emphasized several important factors to ensure a valid implementation of the changing criterion design: “Successful implementation of the changing criterion design requires particular attention to three design factors: length of baseline and treatment phases, magnitude of changes in the criterion, and number of treatment phases or changes in criterion. All phases should be long enough to ensure that successive changes in a therapeutic direction are not naturally occurring due to either historical, maturational, or measurement factors (see Campbell and Stanley, 1963). In addition, treatment phases should differ in length, or if of a constant length, should be preceded by a baseline phase longer than each of the separate treatment phases. This is to ensure that stepwise changes in the rate of the target behavior are not occurring naturally in synchrony with criterion changes” (p. 530).

Klein et al. recommended incorporating “mini-reversals” into the changing criterion design [32]. Such a reversal entails reverting to a previous criterion. For example, if the daily caloric intake for an obese person has been reduced by 200 and 400 calories per day in the first two phases compared to mean caloric intake during the baseline measures, a mini-reversal would entail going back to the 200 calories phase. In actual research practice, such reversals depend, of course, on ethical and practical considerations. If ethically and practically feasible, such reversals can greatly strengthen the demonstration of experimental control over the dependent variable. Klein et al. further recommended that at least three criterion changes should be implemented for repeated observation of intervention effectiveness [32]. Regarding the minimum number of data points required per phase, clear guidelines are still lacking for the changing criterion design. Given the phase structure of the changing criterion design, a reasonable recommendation might be at least three and preferably five data points. The effectiveness of an intervention is demonstrated with a changing criterion design, when the dependent variable consistently changes to criterion levels set by the researcher [66]. In the range-bound version of the changing criterion design [33], the researcher sets a range of acceptable occurrences of the dependent variable instead of a single criterion. Barker et al. gave an example of an injured athlete in rehabilitation [43]. To prevent overtraining and the likelihood of reinjury, it may be useful to place an upper limit on the number of training sessions per week. Similarly, to prevent stagnation in the rehabilitation process, it may be useful to set a lower limit on the acceptable number of weekly training sessions. If the athlete trains no more than the upper limit criterion and no less than the lower limit criterion, then the intervention leads to an acceptable amount of exercising. Thus, the only difference between the classical changing criterion design and the range-bound changing criterion design is that in the former a single-point criterion is set that has to be met by the participant while in the latter an acceptable range is specified.

**Figure 7:**
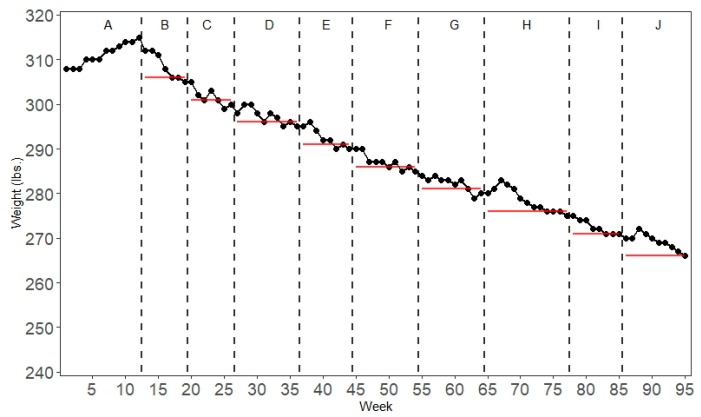
Example of a changing criterion design. The red horizontal lines indicate the criterion in each phase. Data from Singh et al. [67].

Figure 7 shows the results of a changing criterion design using a mindfulness-based health wellness program to reduce the weight of a morbidly obese man [67]. After an initial baseline phase of 12 weeks, during which the subject’s weight was recorded, the intervention was introduced consisting of physical exercise, a food awareness program, mindful eating to manage rapid eating, visualizing and labeling hunger, and a mindfulness procedure as a self-control strategy. Adherence to the physical exercise program resulted in a reward in the form of purchasing an item from the subject’s wish list of reinforcers. During the baseline and all the intervention phases, the subject’s weight was recorded weekly. After the baseline phases, the criterion for each consecutive phase was to lose five pounds. The criterion changed after three successful measures (i.e., the subject’s weight equaled or was below the criterion). Due to the nature of the experiment, ethical considerations rendered it impossible to incorporate a reversal. Neither did the researchers vary the magnitude of criterion changes between phases in consideration with the patient to keep him motivated. In total, the researchers applied the intervention for 270 weeks. To be able to present all the data in a single time-series graph, Figure 7 only shows the data for the first 95 weeks.

Similar to the phase designs, all the data in a changing criterion design are plotted as a single continuous time-series. It is customary to label each phase chronologically in alphabetical order. A visual inspection of the graphed data reveals that the participant lost weight continuously during the course of the experiment. However, it can also be seen that there are several data points in each phase that clearly deviate from the criterion.

Specific randomization schemes for changing criterion designs have only recently been proposed by Onghena et al. [7] and Ferron et al. [68]. Similar to phase designs and multiple baseline designs, a changing criterion design is not eligible for an unrestricted randomization scheme. The specific structure of the changing criterion design has to be taken into account when determining the randomization scheme. The specific structure of the changing criterion design needs to be preserved with its successive phases and criteria when constructing the reference distribution [7]. Another factor that has to be taken into account is that Singh et al. determined that the subject had to record the criterion weight for three weeks before changing the criterion and moving to the next phase [67].

To introduce an element of randomization, we constructed a randomization scheme under the assumption that the researchers determined a priori that the phase change occurs randomly within the next two weeks after the criterion weight had been recorded for at least three consecutive weeks. This leaves us with two possible phase change moments per phase: weeks 19 and 20 in phase B, weeks 26 and 27 in phase C, and so on. There are 256 (calculated by $2^{8}$) possibilities to assign the eight phase change moments in this way.

However, this does not take into account the baseline phase as there is no criterion present in the baseline phase. The different possibilities for incorporating the baseline phase in the randomization procedure and calculation of the test statistic are discussed in Onghena et al. [7]. One possibility would be to drop the baseline measures. As this would result in a loss of possibly valuable information, we do not recommend this option. Another option would be to select a score based on the subject’s characteristics. For example, the subject recorded a weight of 308 pounds when entering the study. One might argue that this would be a sensible criterion under the assumption that the subject will not gain weight. However, this does not take into account how the data pattern in the baseline phase evolves over time and basing a criterion on a single data point seems arbitrary given that this data point might be an outlier. Therefore, we followed the recommendation to take the median value of the baseline phase (311 lbs.) as a criterion [7].

Still, the question—which possible phase change moments to identify for the change from the A- to the B-phase—remains. Given that there are two possible phase change moments in the other phases, we might follow the same logic for the baseline phase, so that in total there are 512 (calculated by $2^{9}$) randomizations. If Singh et al. had incorporated an element of randomization in the planning phase of the experiment, the B-phase might have started randomly after at least five weeks of baseline measures (cf. earlier discussion on the minimum phase length required to meet the evidence standards). Below is a non-exhaustive list of the 512 possible phase change moments for illustrative purposes (the experiment that has actually been carried out is marked in bold):

12, 19, 26, 36, 44, 54, 64, 77, 85
12, 19, 26, 37, 44, 55, 64, 77, 85
12, 20, 26, 36, 44, 54, 64, 77, 85
      …
13, 20, 26, 37, 44, 54, 64, 77, 86
13, 20, 27, 36, 45, 54, 65, 77, 86
**13, 20, 27, 37, 45, 55, 65, 78, 86**.

**Figure 8:**
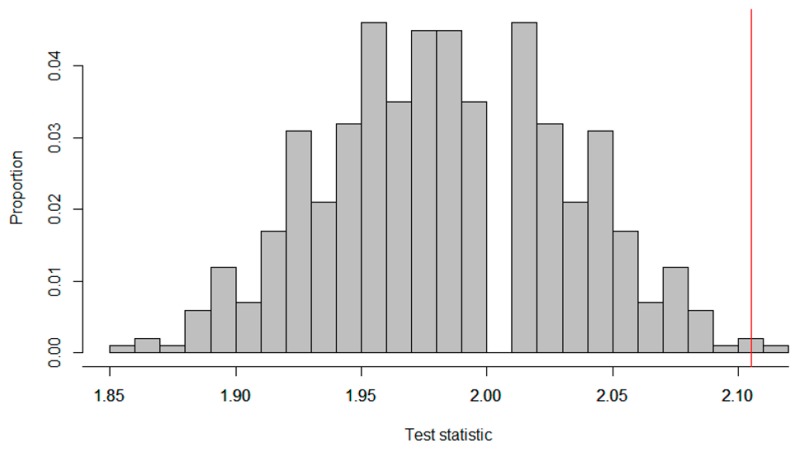
Reference distribution of test statistics for a changing criterion design by Singh et al. [67].

To quantify the intervention effectiveness, we chose the mean absolute deviation as a test statistic [7]. The mean absolute deviation equals the sum of the absolute differences between each individual data point and the criterion within that phase divided by the total number of data points N, which can be written as: $\frac{\sum \left|C_{i}-m_{ij}\right|}{N}$, where C_i_ stands for the criterion in phase i and m_i_ stands for the jth measurement in phase i. The observed test statistic for the data in Figure 7 equals 2.11, meaning that, on average, the subject’s recorded weight deviated 2.11 lbs. from the criterion. For the mean absolute deviation, lower scores indicate a better match between the scores and the criteria. Thus, a score of zero for the absolute mean deviation would indicate a perfect match between the scores and the criterion for all the measurements. Figure 8 shows the distribution of test statistics for all the possible randomizations.

Since for the mean absolute deviance smaller values indicate better adherence to the criterion, we had to look at the left tail of the distribution to calculate the *p*-value. There are 511 randomizations in the distribution that would have led to a lower mean absolute deviance. The *p*-value thus equals 0.998 (calculated by $\frac{511}{512}$), and we did not reject the null hypothesis that the treatment is ineffective.

## 8. Discussion

In the present paper, we showed how randomized SCEDs can be utilized in healthcare research to find individually tailored interventions. For each design type of SCEDs, we presented published studies from the healthcare literature, illustrated how an element of randomization can be incorporated, and how the obtained data can be analyzed by means of visual analysis, effect size measures, and randomization tests. We put the emphasis on the randomization tests because they are a flexible and versatile data analysis technique that can be adapted to many situations encountered in applied research. This emphasis on the randomization tests, however, does not mean that the obtained *p*-value is an all-or-nothing indicator of intervention effectiveness. Visual analysis and effect size calculation, as well as qualitative data, should be considered when judging the success of an intervention for the patient. For example, the *p*-value for the changing criterion design example was nearly 1. At the same time, visual analysis indicated that the patient continuously lost weight throughout the course of the experiment. The patient deviated on average only a bit over 2 lbs. from the criteria. Even though the randomization test indicated a non-significant treatment effect, the weight loss of the patient throughout the experiment can increase his quality of life and overall health. Therefore, we always recommend an integrated approach to analyzing data obtained through SCEDs.

It should be noted that some of the example data sets used in this paper did not incorporate an element of randomization in the planning phase of the study. The analysis of the changing criterion design in particular was loaded with heavy assumptions that were not met in the actual design of the study and the randomization test was calculated only on a subset of data from the original study. However, at the same time, this enabled us to illustrate a possible randomization procedure for the changing criterion design if the researchers incorporated an element of randomization in the planning phase of the study. The randomization tests for the changing criterion design and the ABAB design were therefore carried out under the assumptions that an element of randomization was incorporated a priori and that the experiments as they were carried out were actually chosen at random from all possible randomizations. Alternative ways of analyzing SCED data by means of masked graphs have been proposed for situations, in which the randomization procedure has not (entirely) been determined a priori [69,70]. If the results are analyzed by means of a randomization test when the randomization assumption has not been met, the Type I error might deviate from the predetermined α [71].

An important consideration when conducting any kind of significance testing is the power of the test to detect a treatment effect. The power of randomization tests for phase designs varies as a function of both the number of phases and the number of observations per phase [72]. For multiple baseline designs, it has been found that the power of randomization tests depends, among other things, on the between-case stagger separation, meaning that if the introduction of the intervention is further apart from one case to the next, the power increases [30]. Another important factor in the power of randomization tests for multiple baseline designs is the number of cases, behaviors, or settings under investigation. The power increases considerably when comparing at least three different cases, behaviors, or settings with at least 20 measurements each [18]. For the alternating treatments designs, the power of randomization tests depends largely on the number of observations and the number of permissible successive observations under the same treatment [18]. Research on the specific factors influencing the power of randomization tests for the changing criterion designs is still needed. For SCED randomization tests in general, the lowest possible *p*-value is the inverse of the number of possible randomizations. For the alternating treatments design example, the lowest possible *p*-value was: $\frac{1}{14}=0.07$. When less than 20 randomizations are possible, a randomization test has zero power at a conventional level α of 0.05 [73]. Conversely, as was the case in the multiple baseline design example, the number of possible randomizations can be so high that it becomes computationally unfeasible to calculate the exact *p*-value. As shown, in such cases, a Monte Carlo random sampling procedure can be employed to approximate the exact *p*-value.

Another important consideration in the analysis of SCED data is the choice of data aspects that are of interest to the researcher. Widely accepted guidelines regarding the conduct and analysis of SCEDs recommend inspecting six data aspects: level, trend, variability, overlap, immediacy of the effect, and consistency of data patterns [50]. In the present paper, we focused only on the data aspect level for illustrative purposes. A procedure for assessing all the six data aspects simultaneously through multiple randomization tests has been proposed by Tanious et al. [48]. We do not recommend isolating one data aspect and base a conclusion regarding the effectiveness of an intervention on that data aspect alone. A user-friendly web-based application, where the analyses for phase designs, multiple baseline designs, and alternation designs can be executed, is available at https://tamalkd.shinyapps.io/scda/ [74]. Generic R-code for analyzing changing criterion designs is available in [7]. Further discussion of randomization tests for phase and alternation designs and available software for analyzing these designs are available in a study of Heyvaert and Onghena [75].

## 9. Conclusions

Randomized SCEDs are valid alternatives to large-group studies for applied healthcare professionals, especially when patient symptoms are highly idiosyncratic in nature. Randomization tests allow for powerful inferences regarding treatment effectiveness based on the random assignment procedure actually used in the experiment.

## Figures and Tables

**Table 1:** Overview of single-case experimental design (SCED) options with references for further reading.

Type of SCEDs	Design examples	Key references
Phase designs	AB ABA ABAB ABAC ABACA	[10,14,15]
Alternation designs	Completely randomized design Randomized block design Alternating treatments design	[16,20] [21,22,23,24] [25,26,27,28]
Multiple baseline design	Across participants Across outcomes Across settings	[29,30]
Changing criterion design	Single-point criteria Range-bound criteria	[31,32] [33,34]
